# Supplementary material for: A mouse tissue transcription factor atlas
Source: Nat Commun. 2017 Apr 21;8:15089. doi: 10.1038/ncomms15089 (PMC5413951; doi:10.1038/ncomms15089)
Supplement: Supplementary Information — Supplementary Figures. [file ncomms15089-s1.pdf]

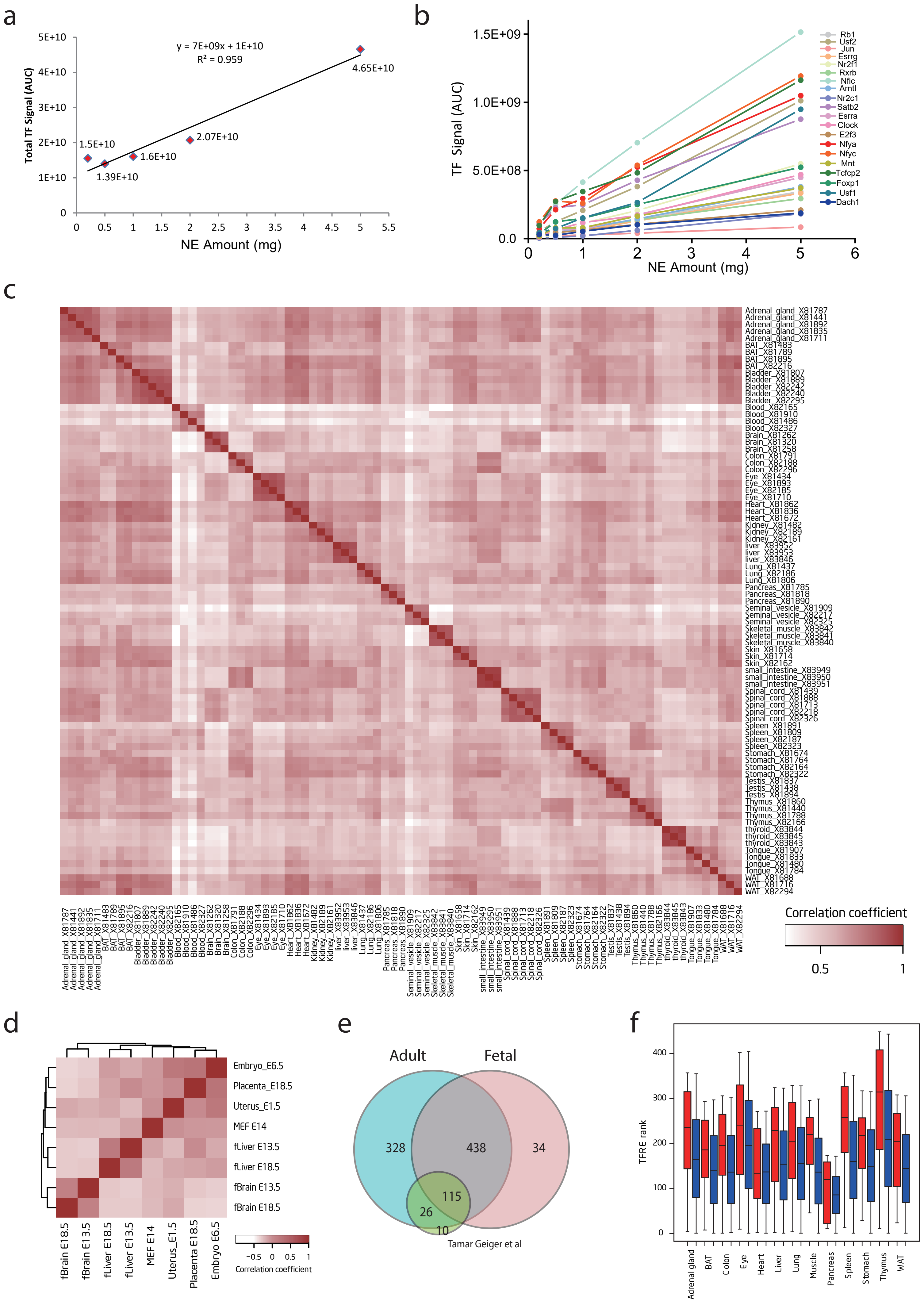
a
Total TF Signal (AUC)
NE Amount (mg)
y = 7E+09x + 1E+10
R² = 0.959
4.65E+10
2.07E+10
1.5E+10
1.6E+10
1.39E+10
0
1E+10
2E+10
3E+10
4E+10
5E+10
0 0.5 1 1.5 2 2.5 3 3.5 4 4.5 5 5.5
b
TF Signal (AUC)
NE Amount (mg)
0.0
5.0E+08
1.0E+09
1.5E+09
0 1 2 3 4 5 6
Rb1
Usf2
Jun
Esrrg
Nr2f1
Rxrb
Nfic
Arntl
Nr2c1
Satb2
Esrra
Clock
E2f3
Nfya
Nfyc
Mnt
Tcfcp2
Foxp1
Usf1
Dach1
c
Adrenal_gland_X81787
Adrenal_gland_X81441
Adrenal_gland_X81892
Adrenal_gland_X81835
Adrenal_gland_X81711
BAT_X81483
BAT_X81789
BAT_X81895
BAT_X82216
Bladder_X81807
Bladder_X81889
Bladder_X82242
Bladder_X82240
Bladder_X82295
Blood_X82165
Blood_X81910
Blood_X81486
Blood_X82327
Brain_X81262
Brain_X81320
Brain_X81258
Colon_X81791
Colon_X82188
Colon_X82296
Eye_X81434
Eye_X81893
Eye_X82185
Eye_X81710
Heart_X81862
Heart_X81836
Heart_X81672
Kidney_X81482
Kidney_X82189
Kidney_X82161
liver_X83952
liver_X83953
liver_X83846
Lung_X81437
Lung_X82186
Lung_X81806
Pancreas_X81785
Pancreas_X81818
Pancreas_X81890
Seminal_vesicle_X81909
Seminal_vesicle_X82217
Seminal_vesicle_X82325
Skeletal_muscle_X83842
Skeletal_muscle_X83841
Skeletal_muscle_X83840
Skin_X81658
Skin_X81714
Skin_X82162
small_intestine_X83949
small_intestine_X83950
small_intestine_X83951
Spinal_cord_X81439
Spinal_cord_X81888
Spinal_cord_X81713
Spinal_cord_X82218
Spinal_cord_X82326
Spleen_X81891
Spleen_X81809
Spleen_X82187
Spleen_X82323
Stomach_X81674
Stomach_X81764
Stomach_X82164
Stomach_X82322
Testis_X81837
Testis_X81438
Testis_X81894
Thymus_X81860
Thymus_X81440
Thymus_X81788
Thymus_X82166
thyroid_X83844
thyroid_X83845
thyroid_X83843
Tongue_X81907
Tongue_X81833
Tongue_X81480
Tongue_X81784
WAT_X81688
WAT_X81716
WAT_X82294
Correlation coefficient
0.5
1
d
Embryo_E6.5
Placenta_E18.5
Uterus_E1.5
MEF E14
fLiver E13.5
fLiver E18.5
fBrain E13.5
fBrain E18.5
-0.5 0 0.5 1
Correlation coefficient
e
Adult
Fetal
328
438
34
115
26
10
Tamar Geiger et al
f
TFRE rank
0
100
200
300
400
Adrenal gland
BAT
Colon
Eye
Heart
Liver
Lung
Muscle
Pancreas
Spleen
Stomach
Thymus
WAT

Supplementary Figure 1. Correlation of the TF profiles between biological replicates and comparison with profiling data. (a) Quantitative feasibility and linearity of catTFRE strategy evaluated by dilution analysis. Different amounts of NE extracted in brain were used as shown. Total peptide AUC (area under curve) was calculated. (b) Quantitative feasibility and linearity of individual DBTFs. (c) Heat map of the TF DNA-binding abilities in the NEs (rows) from 84 samples of 24 adult mouse tissues. (d) Heat map of the TF DNA-binding abilities in the NEs (rows) from 8 fetal mouse tissues. (e) Venn diagram shows our data covers most of TFs that Geiger et al identified. (f) TFs detected in profiling data are in higher abundance part of TFRE data. Y-axis showed TF rank in TFRE data. Red boxes are overlapped TFs by profiling data and TFRE data.

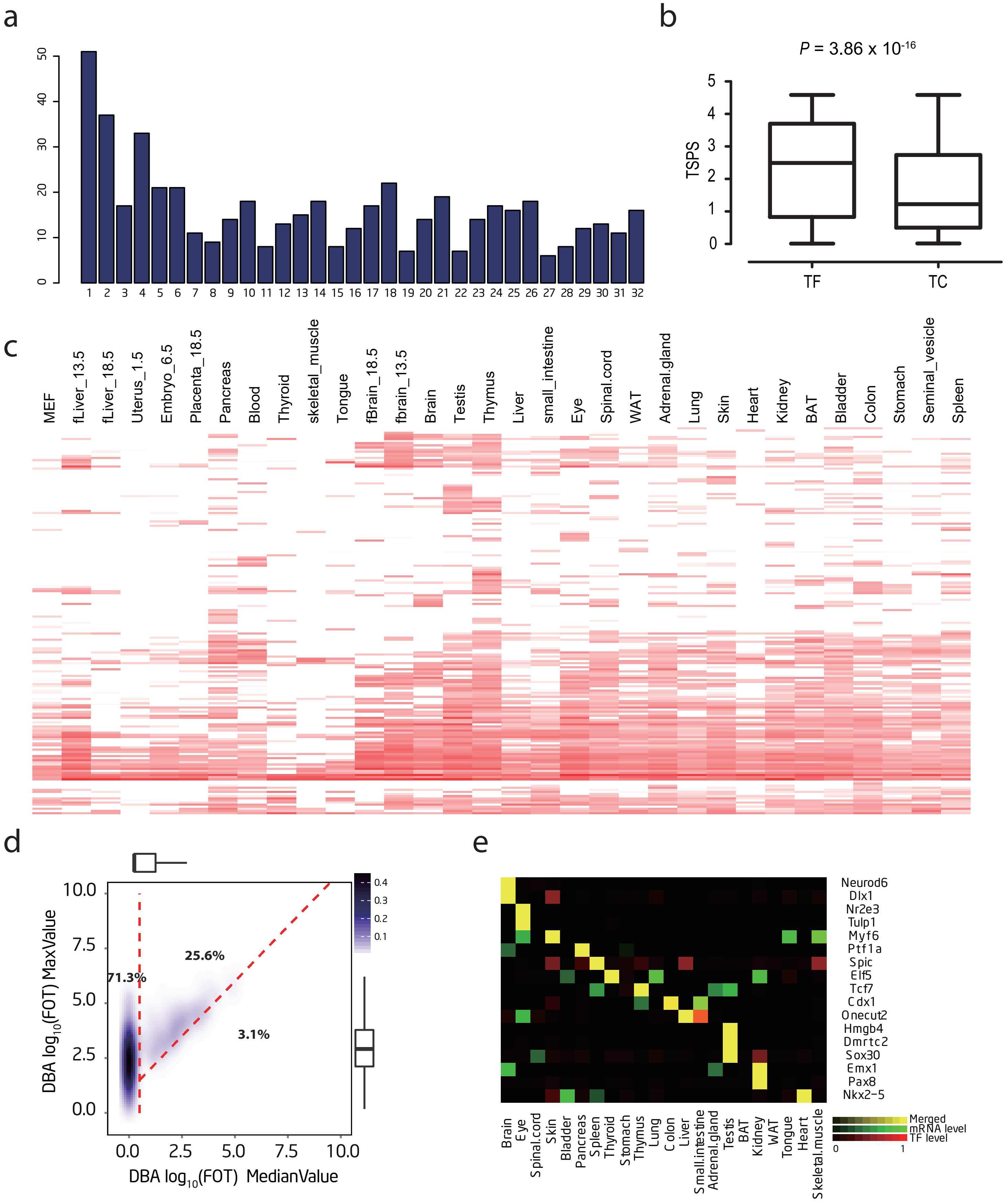

Supplementary Figure 2. TC identifications and tissue-type restricted TF expressions in 32 mouse tissues.

(a) Number of tissues in which the TCs are expressed. (b) Comparison of TSPS between TF and TC. (c) Heatmap for TCs in the 32 mouse tissues. (d) Distributions of ubiquitous and ttrTFs and their relative abundances in 24 adult tissues. The abundance of the TFs spans almost 7 orders of magnitude. The names of the top 3 most abundant TFs are listed. (e) Validation of 17 ttrTFs by qPCR using actin as a control in adult tissues. Red color: TF DNA binding activity; green color: mRNA; yellow color: merging of the mRNA and TF DNA binding activities.

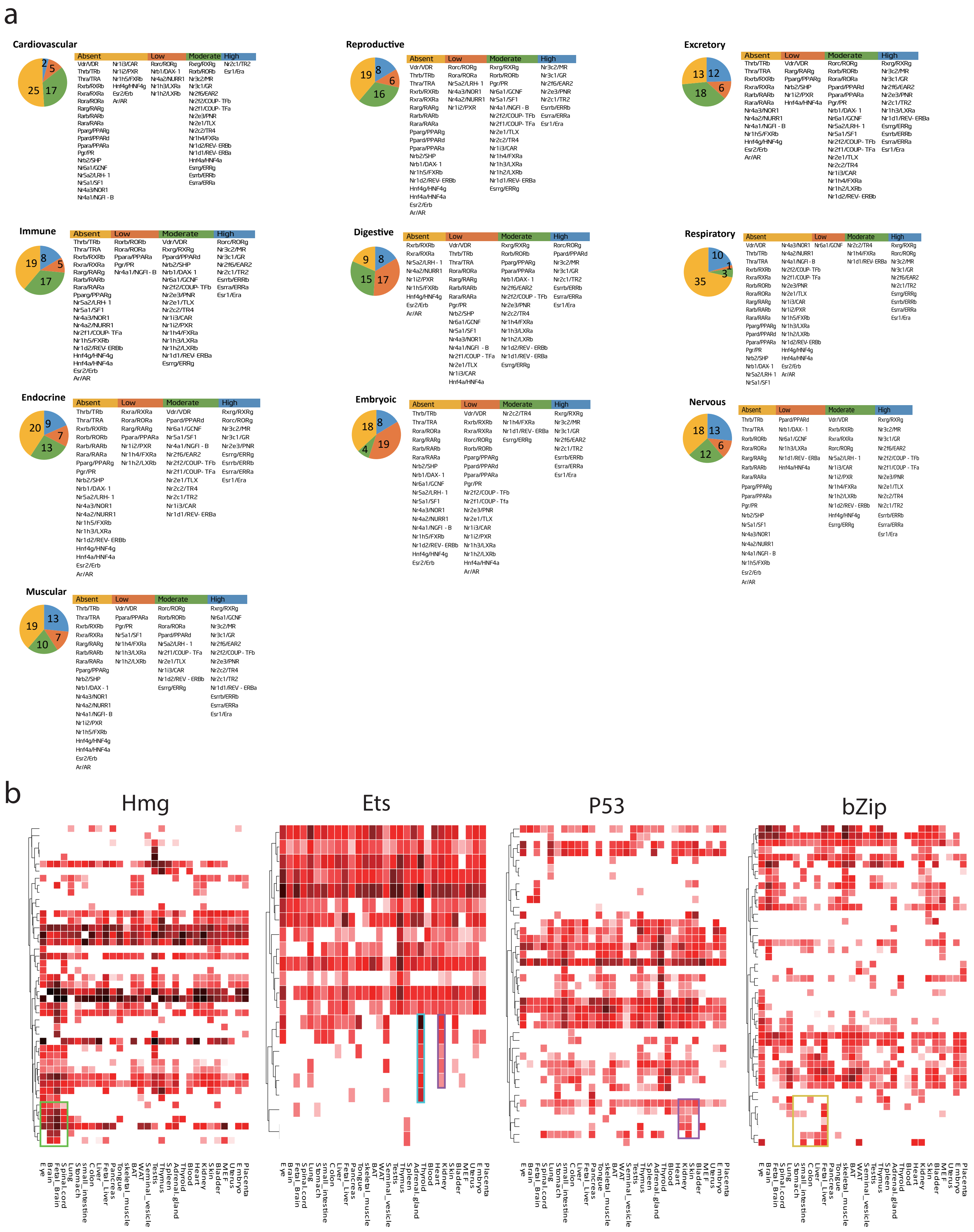

Supplementary Figure 3. Distributions of NRs in ten physiological systems.

(a) The NR expression levels in different physiological systems are indicated by the pie charts, and their names are listed in the tables to the right. The tissue systems were categorized as described in Figure 1. (b) Unsupervised hierarchical clustering of other TF families. Blocks with different colors represent different physiological systems.

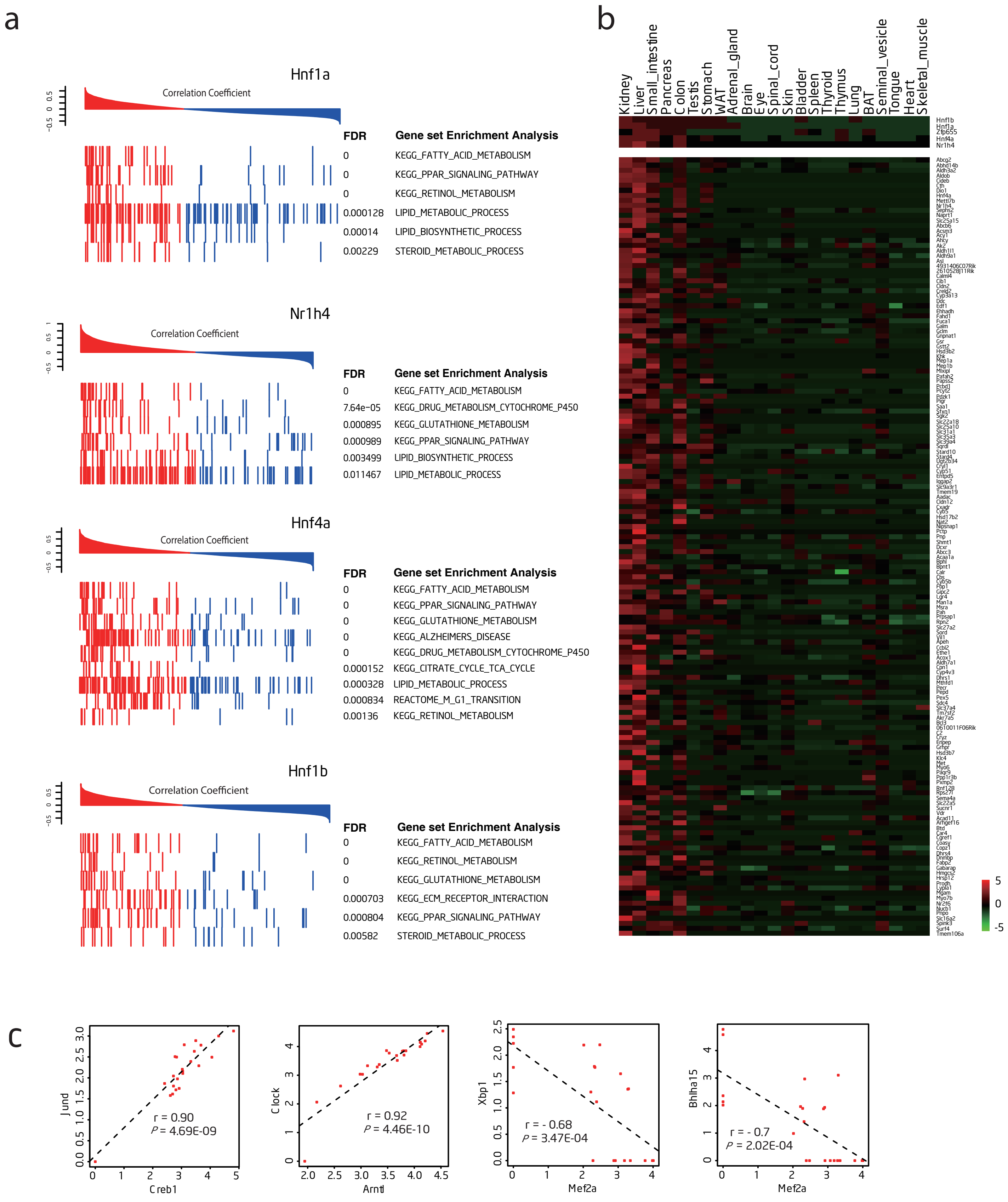

Supplementary Figure 4. DBTF co-expression network in 24 adult tissues.

(a) GSEA terms of other four TFs in Module #12 suggest their functions in metabolism. (b) Gene sets associated with the TFs in nine small modules. For example, Module #12 contained 5 TFs, and the mRNAs in the leading edge of the GSEA for the 5 TFs were enriched for both metabolism and immunity. (c) Correlations of individual TFs. The test for the association between paired samples was based on Pearson's product moment correlation coefficient.

a

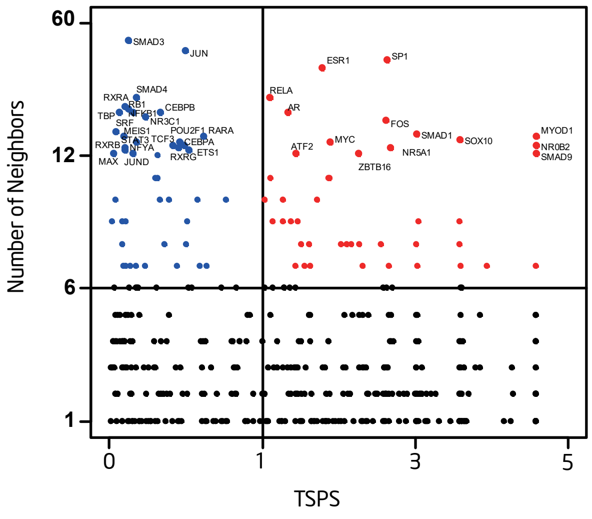

b

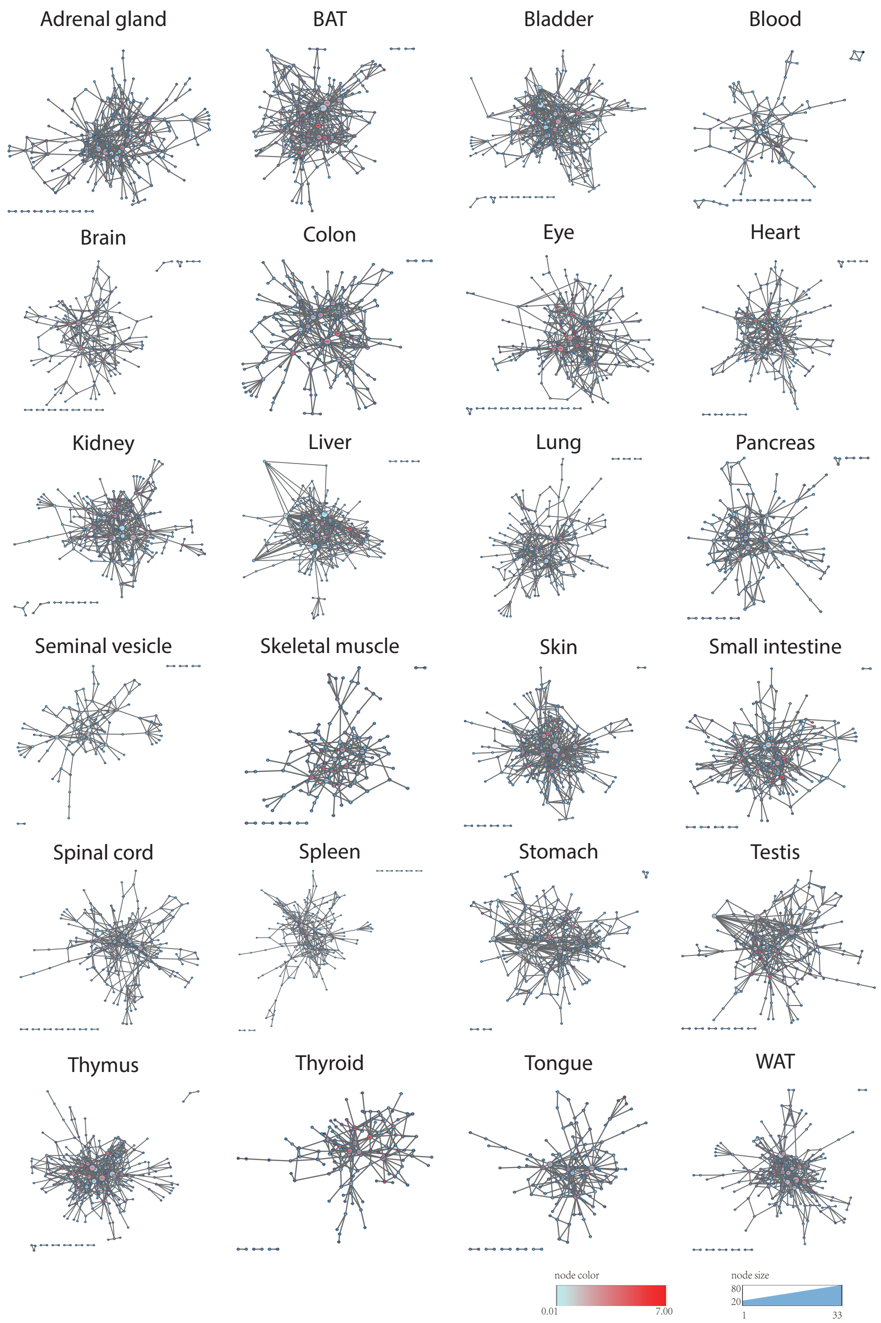

c

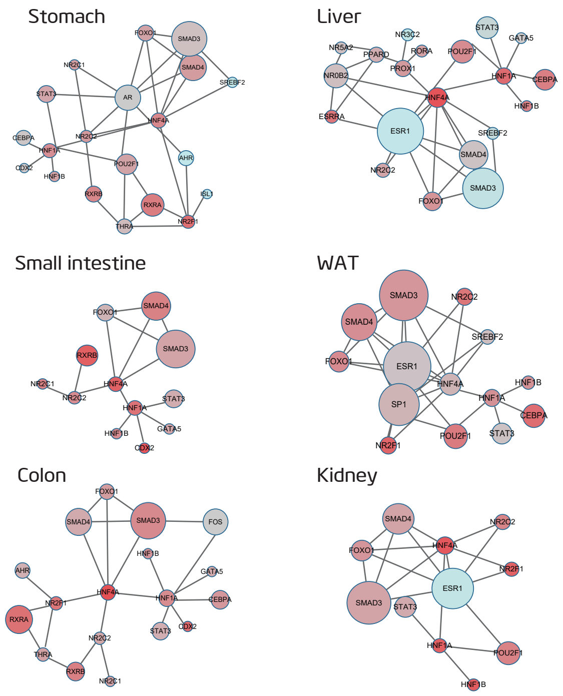

d

Respiratory system	
vasculature development	1.86E-13
blood vessel development	3.73E-13
cell adhesion	4.20E-07
angiogenesis	3.46E-07
extracellular matrix organization	1.14E-06
respiratory system development	2.37E-04
morphogenesis of a branching structure	2.49E-04
circulatory system process	2.89E-04
lung development	2.89E-04

Cardiovascular system	
heart development	1.21E-16
cardiac muscle tissue development	1.89E-10
myofibril assembly	2.54E-09
muscle cell differentiation	1.97E-08
actomyosin structure organization	4.74E-08
striated muscle tissue development	4.23E-07
muscle organ development	3.92E-07
cardiac cell differentiation	7.01E-06

Excretory system	
ion transport	2.70E-10
epidermal cell differentiation	2.34E-04
transmembrane transport	3.50E-04
epidermis development	6.21E-04

Digestive system	
oxidation reduction	2.46E-12
steroid metabolic process	4.49E-10
coagulation	5.19E-07
regulation of body fluid levels	4.17E-07
lipid transport	1.42E-06
wound healing	4.38E-05
bile acid metabolic process	1.36E-03
complement activation	1.37E-03

Reproductive system	
sexual reproduction	7.54E-72
gamete generation	1.50E-56
reproductive process in organism	1.04E-54
spermatogenesis	7.80E-54
reproductive cellular process	2.29E-27
reproductive developmental process	6.21E-22
meiosis	9.92E-22
fertilization	2.37E-19

Endocrine system	
secretion by cell	6.58E-04
secretion	6.53E-04
antigen processing and presentation	9.76E-04
synaptic vesicle transport	9.80E-04
exocytosis	6.23E-03

Nervous system	
neuron differentiation	2.4E-03
transmission of nerve impulse	1.91E-02
spinal cord development	2.17E-02

Muscular system	
skeletal system development	8.10E-11
pattern specification process	4.67E-09
skeletal system morphogenesis	2.39E-08
regionalization	2.30E-08
muscle contraction	2.81E-05
muscle organ development	4.51E-05
muscle system process	6.85E-05

Immune system	
cell cycle	1.81E-41
mitosis	1.15E-28
lymphocyte activation	2.06E-16
leukocyte activation	6.54E-16
positive regulation of immune system process	1.02E-13
immune response	4.19E-10

Embryoic tissues	
neuron differentiation	4.80E-19
cell morphogenesis in differentiation	3.18E-09
cell part morphogenesis	3.18E-09
cell projection organization	3.13E-09

Supplementary Figure 5. TF interaction network and functions of differentially expressed systemic TFs.

(a) Scatterplot of TSPS (x-axis) versus the number of neighbors (y-axis). Red points are defined as specifier hubs, and blue points are defined as facilitator hubs. (b) PPIs of all TFs in the 24 tissues. The colors show the DNA-binding abilities in the NEs, and the node size indicates the number of PPIs of the TF. (c) Hnf4a TF interaction network in stomach, liver, small intestine, WAT, colon and kidney. (d) The GO terms in each system were enriched using the genes that were regulated by two or more differentially expressed systemic TFs.

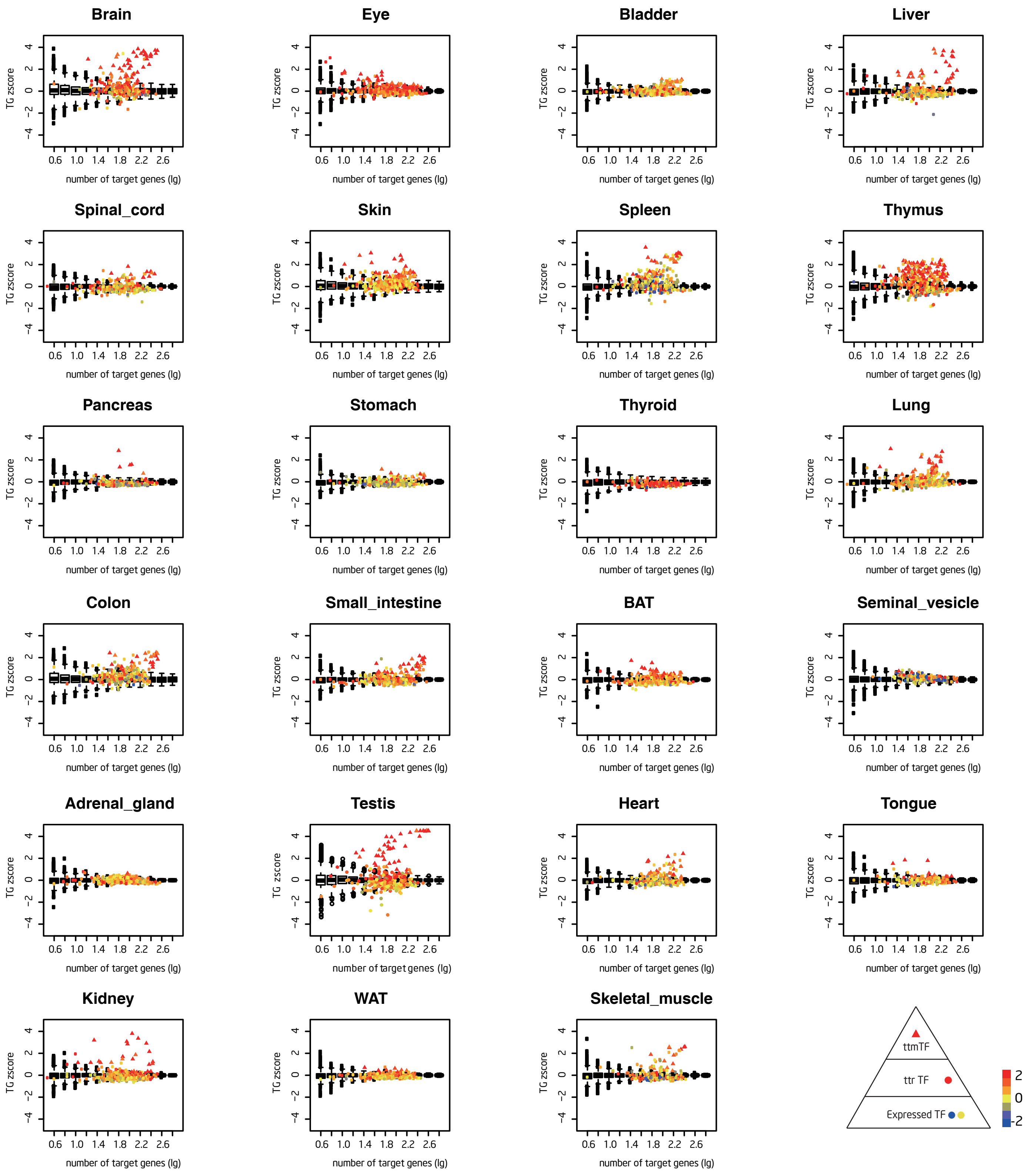

Supplementary Figure 6. ttmTFs for each tissue.

ttmTFs are TFs with high Z-scores (>1) and TG Z-scores that are higher than those for random data.

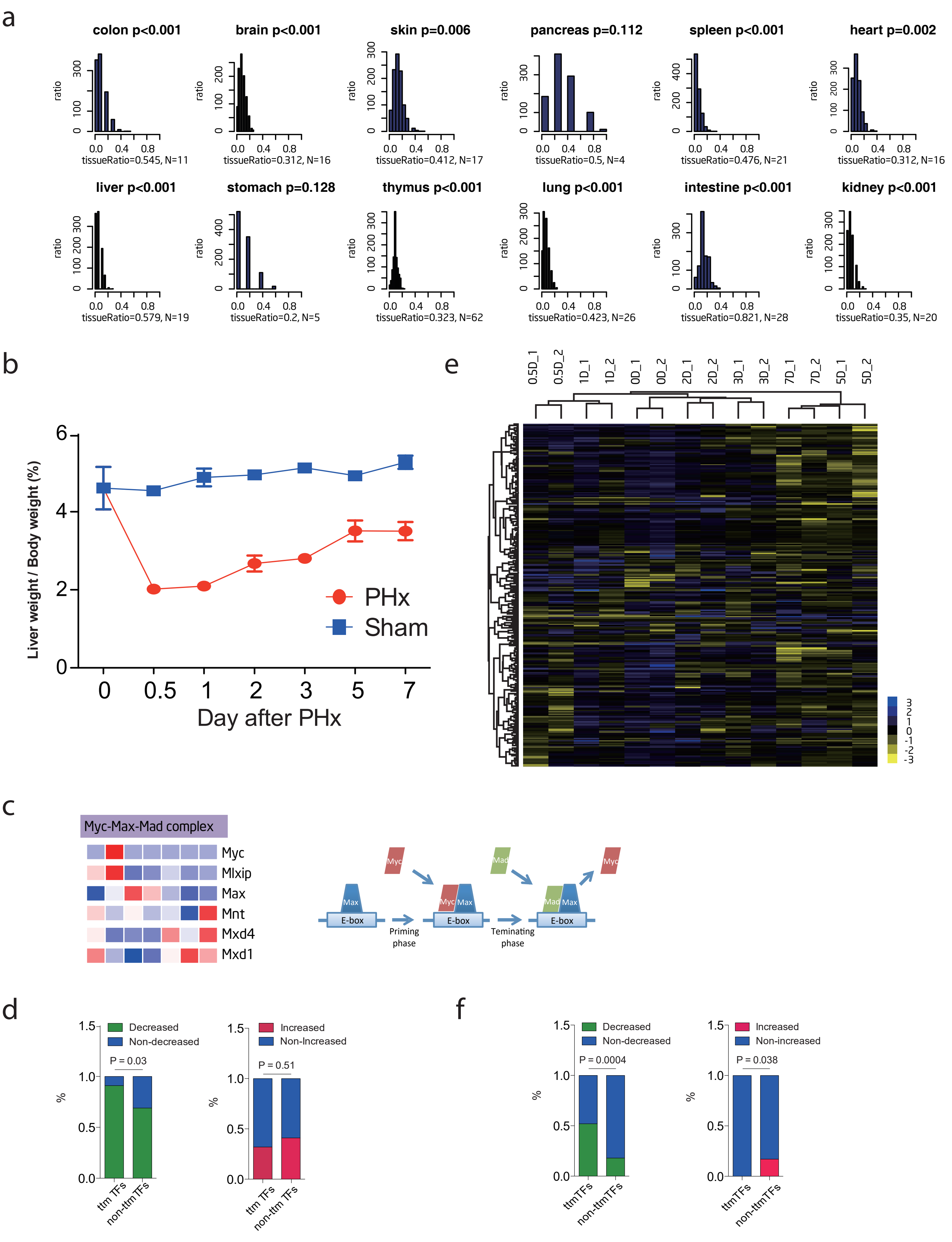

a
colon p<0.001
tissueRatio=0.545, N=11
brain p<0.001
tissueRatio=0.312, N=16
skin p=0.006
tissueRatio=0.412, N=17
pancreas p=0.112
tissueRatio=0.5, N=4
spleen p<0.001
tissueRatio=0.476, N=21
heart p=0.002
tissueRatio=0.312, N=16
liver p<0.001
tissueRatio=0.579, N=19
stomach p=0.128
tissueRatio=0.2, N=5
thymus p<0.001
tissueRatio=0.323, N=62
lung p<0.001
tissueRatio=0.423, N=26
intestine p<0.001
tissueRatio=0.821, N=28
kidney p<0.001
tissueRatio=0.35, N=20
ratio
b
Liver weight / Body weight (%)
Day after PHx
PHx
Sham
0 0.5 1 2 3 5 7
e
0.5D_1 0.5D_2 1D_1 1D_2 0D_1 0D_2 2D_1 2D_2 3D_1 3D_2 7D_1 7D_2 5D_1 5D_2
3 2 1 0 -1 -2 -3
c
Myc-Max-Mad complex
Myc
Mlxip
Max
Mnt
Mxd4
Mxd1
E-box
Priming phase
Teminating phase
Mad
d
Decreased
Non-decreased
P = 0.03
ttm TFs
non-ttm TFs
Increased
Non-Increased
P = 0.51
%
f
Decreased
Non-decreased
P = 0.0004
ttmTFs
non-ttmTFs
Increased
Non-increased
P = 0.038

Supplementary Figure 7. The tsDMRs of ttmTF promoters and additional details for 2 biological repeats of the liver PHx time course data.

(a) The ttmTF promoters in a certain tissue were enriched in that tissue's tsDMRs. Almost all tissues had an enrichment p value <0.05, except for brain (p=0.104), kidney (p=0.11) and heart (p=0.106). (b) Liver/body weight ratios for 2 biological repeats of the liver PHx time course data. Data are represented as mean ± SEM. (c) Myc/Max/Mad network in regulating liver regeneration. (d) Enrichment of down-regulated and up-regulated ttmTFs after PHx. The ttmTFs were significantly decreased compared with the non-ttm TFs after PHx. Fisher's exact test was used to test for significance. (e) Hierarchical clustering of the TF patterns during liver regeneration. Two biological repeats for each time point were co-clustered. (f) TF's target genes in profiling data were used to predict change in TFs. And enrichment of down-regulated ttmTFs was also detected in the PHx time-course data, while no enrichment for up-regulated ttmTFs. Fisher's exact test was performed to test for significance.
